# Overcoming the effects of rogue taxa: Evolutionary relationships of the bee flies

**DOI:** 10.1371/currents.RRN1233

**Published:** 2011-05-05

**Authors:** Michelle D. Trautwein, Brian M. Wiegmann, David K Yeates

**Affiliations:** ^*^Department of Entomology, North Carolina State University, Raleigh NC 27695 and ^‡^CSIRO Ecosystem Sciences, Australian National Insect Collection, PO Box 1700 Canberra ACT 2601

## Abstract

Bombyliidae (5000 sp.), or bee flies, are a lower brachyceran family of flower-visiting flies that, as larvae, act as parasitoids of other insects. The evolutionary relationships are known from a morphological analysis that yielded minimal support for higher-level groupings. We use the protein-coding gene CAD and 28S rDNA to determine phylogeny and to test the monophyly of existing subfamilies, the divisions Tomophtalmae, and ‘the sand chamber subfamilies’. Additionally, we demonstrate that consensus networks can be used to identify rogue taxa in a Bayesian framework. Pruning rogue taxa post-analysis from the final tree distribution results in increased posterior probabilities. We find 8 subfamilies to be monophyletic and the subfamilies Heterotropinae and Mythicomyiinae to be the earliest diverging lineages. The large subfamily Bombyliinae is found to be polyphyletic and our data does not provide evidence for the monophyly of Tomophthalmae or the ‘sand chamber subfamilies’.

**Introduction**

Bombyliidae are among the most species-rich (>5000 sp.) and morphologically diverse families of Diptera.  Commonly known as bee flies, they are flower-visiting hymenoptera-mimics as adults and insect parasitoids (ecto-, endo- and hyperparasitoids) as larvae. Bee fly species are known to be parasitoids of both crop pests and beneficial insects, such as Noctuidae and solitary bees ([1]; [2]), yet their economic relevance as pollinators and parasitoids of pests has not been explored.  Their morphological diversity is wide-ranging, exhibited by microbombyliids as small as the head of a pin --and large, speckled-winged anthracines with a wingspan greater than 2 inches.   Though many common bee flies can be recognized by a unique combination of characters, there is not a non-homoplastic, diagnostic synapomorphy uniting all members of the family ([3]). Striking anatomical and species diversity has contributed to the complexity of determining evolutionary relationships among bee flies using morphology alone.

Bombyliids are a relatively early diverging lineage within the large radiation of flies known as Brachycera (short-horned flies).  Many related lineages within lower brachycera are also large-bodied flower-visiting flies. Recent evidence suggests that the orgin and diversification of the lower brachyceran families such as Bombyliidae coincides with the origin of flowering plants approximately 215 MA.  Bee flies have long been considered a member of the superfamily Asiloidea, along with robber flies (Asilidae), mydas flies (Mydidae) and stiletto flies (Therevidae).  Recent molecular analyses conflict in their support for placing the bee flies either within a monophyletic Asiloidea (as the sister group to the remaining asiloids) ([4]) or as the sister group to the asiloids + the higher flies ([5]).  Putative bombyliid fossils have been found that are millions of years older than any other asiloid fossil (Grimaldi, 2005; Wedmann and Yeates, 2008), supporting the idea that bee flies may be the earliest diverging asiloid family.

The classification of bee fly subfamilies and the relationships between these subfamilies has a confounding history.  Yeates (1994) [3] and Hull (1973) [1] provide the most thorough reviews of bee fly classification and phylogenetics.  The two largest bee fly subfamilies, Bombyliinae and Anthracine, include the majority of species and were once the only two subfamilies in the classification ([1] [6]).   Bee fly taxonomy later became 'top heavy with subfamilies' ([1] [3]) including over 23. An early morphology-based phylogenetic analysis of the family done by Muhlenberg (1971) [7] laid the modern ground plan for the higher-level relationships within the family. The only other quantitative analysis completed across the family was done by Yeates in 1994 [3] using morphology, and resulted in the maintenance of 15 subfamilies that were largely established by Hull [1].   Yeates' phylogeny-based classification is the foundation for tests of clade recovery in our molecular study (Fig. 1).

**Figure:**
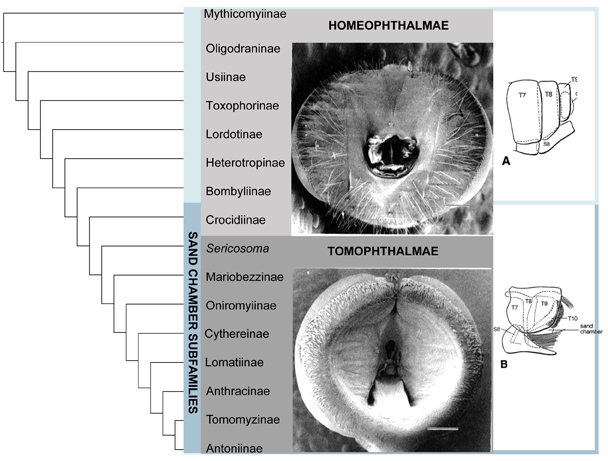
Fig. 1.  Relationships of bee fly subfamilies based on the morphological analysis of Yeates 1994. Bombyliidae are divided into the Homophthalmae and the Tomothalmae based on characters of concavity of the post-occiput and number of occipital foramen.  The sand chamber subfamilies largely correspond to the Tomopthalmae.

Bee flies are currently divided into two major groups, Homeophthalmae and Tomophthalmae [8] [9]. The defining features of Homeophthalmae and Tomophthalmae have been altered over the years, and the current definition was established by Evenhuis (1991) [9].   'Homeophthalmae' is a paraphyletic group that includes a ladder of early diverging bombyliids that have one occipital foramen corresponding to a flat or bulbous post cranium, primarily represented by the large subfamily Bombyliinae (Fig. 1). Tomophthalmae is considered a monophyletic group of more derived bee flies, including the largest subfamily Anthracinae, that possess two occipital foramina and varying degrees of concavity of the postcranium (Fig. 1).   

Another important feature that divides the bombylliids is a female genetalic character called a sand chamber.  The sand chamber allows bee flies to protectively coat their eggs with sand before flicking them onto the substrate in the vicinity of a potential host for their larvae.  Though bee flies can be found in all zoogeographic regions, they are most diverse in arid, sandy regions.  The most species-rich subfamilies possess sand chambers, and it has been hypothesized that this feature ensures a specialization to ground-dwelling hosts that has allowed bee flies to avoid direct competition with more efficient parasitoids, including wasps and tachinid flies [3] [2] [10]. Subfamilies that possess a sand chamber form a monophyletic group, with some examples of secondary loss, previously known as Psammomorphidae, now termed 'sand chamber subfamilies' [3] [7].   This group corresponds to the Tomophthalmae along with their putative closest relatives, Bombyliinae and Crocidiinae (Fig. 1).   

In a recent molecular phylogenetic study of the superfamily Asiloidea, bee flies were disproportionately found to act as rogue taxa [5]. Rogue taxa can be defined as taxa with unstable or varying phylogenetic placement that have a generally negative effect on topological resolution and support values [11]. These effects are particularly detrimental in respect to majority consensus trees (Fig. 2) [12] [13] [14]) and thus the issue of rogue taxa is especially relevant in a Bayesian framework [15] [12].  Bayesian analysis results not in a single estimate of phylogeny, but in a distribution of trees that is most often represented by a majority consensus tree [15] [16]).  Relationships that are prevalent in the tree distribution may not be represented, or may have misleadingly low support in a majority consensus tree if unstable taxa occasionally insert themselves within these common groupings [11] [12] [13] [14].  Nodes that are unsupported in the presence of rogue taxa may be highly supported in their absence [12] [13]. Thus, in phylogenies plagued by limited resolution and low support values, it becomes useful to explore tree topologies to identify and remove rogue taxa . 

**Figure:**
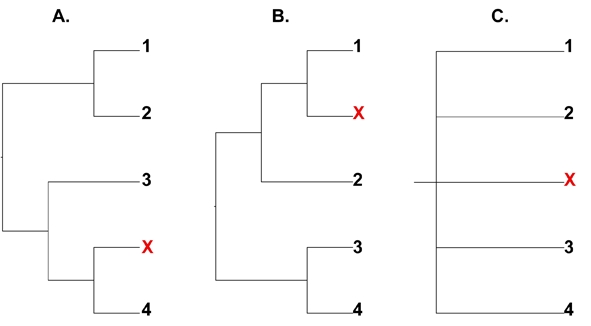
Figure 2.  Majority rule consensus trees can be greatly affected by rogue taxa.  Prevelant relationships may be unresolved or unsupported if interrupted by taxa with unstable placements.  Trees A. and B. are identical but for the placement of a single taxon (x).  C. The shared structure between them is lost in a majority consensus tree.

In practice, the identification of rogue taxa often takes place prior to final analyses through non-quantitative observation of taxa exhibiting multiple placements in preliminary analyses as a result of either changes in parameters, taxon sampling or the use of alternate analytical algorithms.  A more quantitative way to identlfy rogue taxa is to rely on leaf stability measures [17].  A certain measure of instability can be arbitrarily determined and taxa that do not meet the stability cut-off are deemed ‘rogue’ [18] [19] [5].   Consensus networks are a means of providing visual information regarding taxon instability within a set of trees. Consensus networks simultaneously display multiple trees, revealing conflicting splits, and have previously been demonstrated to be useful in displaying disconcordance of gene trees [20], conflict between methods of analysis [21] [22], and in identifying artifactual placements ([22]. In a consensus network, taxa that have multiple attachment points across various trees exhibit reticulation in their nodes [23], and thus can be identified as potentially rogue. 

Once identified, it is common practice for rogue taxa to be simply removed from final analyses and subsequently they often go unreported in published work.  However, including rogue taxa in analyses and then pruning them from the resulting tree (or set of trees) allows them to phylogenetically inform the analysis while avoiding their deleterious effects on resolution and support values [15] [12].  Simulations have shown that pruning them from trees post-analysis more closely reflects the true tree [24] [25] [15].  Though recently developed theoretical methods to rectify the effects of rogue taxa focus on post-analysis ‘leaf-dropping’ methods [15] [12] [13], this practice does not appear to be routine amongst systematists.

Our study is the first to utilize molecular data to address evolutionary relationships across the family Bombyliidae.  Using the nuclear protein-coding gene CAD and 28S ribosomal DNA, we aim to test the monophyly of currently accepted subfamilies, determine the relationships between these subfamilies, and to test the monophyly of the large divisions Tomophthalmae and the sand chamber subfamilies. We examine taxon instability using consensus networks and prune rogue taxa post-analysis in an effort to increase support values.

**Materials and Methods**

*Taxon sampling*

Fifty- nine taxa, including 55 genera, representing 13 out of 15 bee fly subfamilies and four lower brachyceran outgroups, were sampled for CAD and 28S rDNA (approx. 5kb.) (Table 1).  The two subfamilies not represented are small monogeneric subfamilies with limited zoogeographical distribution:  Oligodraninae, includes a small genus restricted to the Palearctic that was previously placed in Phthiriinae or Usiinae [26], [3]; and Oniromyiinae, with only two species, is found only in South Africa.

**Figure:**
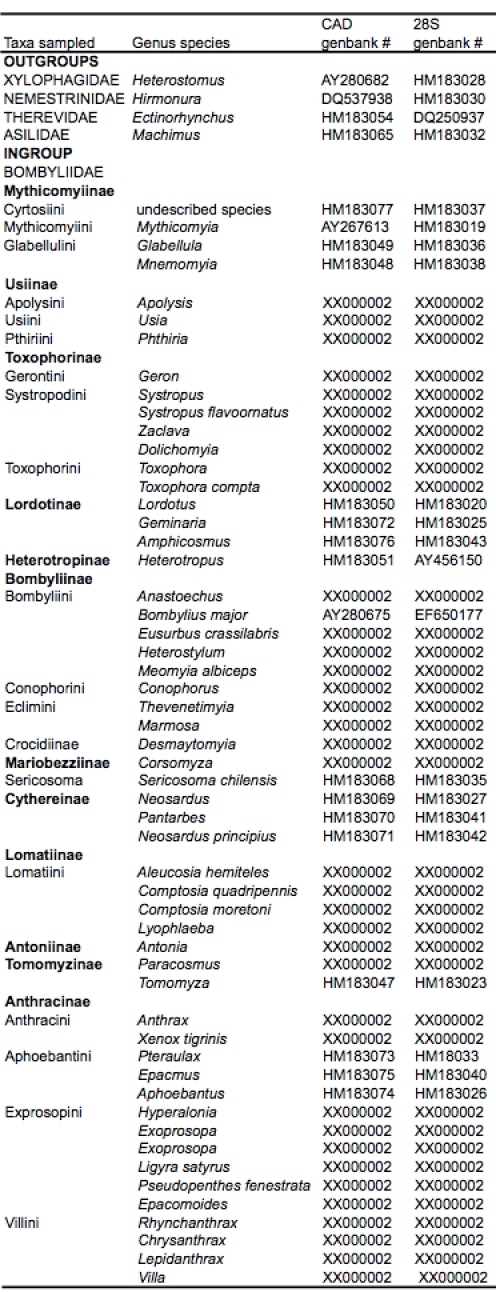
Table 1.  Taxa sampled for analysis with genbank numbers.

*DNA Extraction, Amplification and Sequencing*

Genomic DNA was extracted using the DNeasy DNA extraction kit (QIAGEN Inc., Valencia, CA). The standard protocol was altered by extending the amount of time the specimen was in proteinase K solution to two days in order to effectively break down chitin but to avoid grinding the specimen.  Final elution was reduced to 30 ml to avoid diluting the DNA solution.

Sequence data was collected from all 59 taxa for two nuclear genes, the protein-coding gene CAD (carbamoyl phosphate synthetase-aspartate transcarbamoylase-dihydroorotase) and 28S ribosomal DNA. For CAD, approximately 4000bp from the carbomoylphosphate synthase (CPS) domain of the gene were amplified and sequenced using Diptera-specific degenerate primers designed by Moulton (Moulton and Wiegmann, 2004), in addition to primers tailored specifically to bee fly taxa.   To amplify approximately 1000 bp from the 3’ end of 28S rDNA, we used published Diptera primers [27].    PCR parameters varied for CAD and 28S [5].  PCR products were extracted from low-melt agarose gels and purified with the Qiaquick Gel Extraction kit (Qiagen, Santa Clara, CA).  Big Dye Sequencing kits (Applied Biosystems, Foster City, CA) were used for sequencing reactions and sequencing was completed at the North Carolina State University, Genome Sequencing Laboratory (GSL).  Sequences were contiged and edited using Sequencher 4.1 (Gene Codes Corp., Ann Arbor, MI).

Alignment was carried out manually using Se-Al 2.0 ([28].   CAD was aligned according to its amino acid translation. Introns in CAD, hypervariable regions of 28S, and other positions of ambiguous alignment were removed from the data set.  To detect existing base compositional bias, a chi-square test for homogeneity of base frequencies across taxa was performed for the concatenated CAD+28S data set with (p= 00000) and without the 3^rd^ positions of CAD (p=.99996) using Paup* 4.0b10 [29].  

*Phylogenetic Analyses*

Maximum parsimony (MP), maximum likelihood (ML) and Bayesian (BI) analyses were completed with both genes and of each independent gene.  In addition, we conducted a MP AND BI ‘total evidence’ analysis that included 154 morphological characters from Yeates (1994) [3] combined with our concatenated molecular data (Table 2).  To avoid the effects of saturation and systematic bias due to base composition in the 3^rd^ codon position, all analyses including CAD were completed with and without 3^rd^ positions.

*Parsimony Analyses*

Maximum parsimony analyses were done using Paup* 4.0b10 [29]. Heuristic searches with TBR branch swapping and 500 random addition replicates were completed to find the shortest trees. Node support was obtained by acquiring bootstrap values from heuristic searches of 500 re-sampled data sets and 50 random addition replicates.

*Bayesian Analyses*

An appropriate model of nucleotide evolution, in this case GTR + I + G, was chosen by using Mr. Modeltest for each gene independently [30].  Using Mr.Bayes [31] [32], analyses were conducted for 20,000,000 generations, trees sampled every 1000, with the first 25% discarded as burn-in. Each gene was treated as a separate partition; however when 3rd positions were included, each codon position of CAD was treated as a separate partition. Parameters were unlinked between partitions. Runs were continued for millions of generations after the standard deviation of split frequencies fell below .01.  For our total evidence analysis, we utilized a standard discrete (morphology) model, Markov k [33] for our morphological partition.

*Maximum Likelihood Analyses*

Maximum likelihood analyses were performed using Garli [34] with a GTR+I+G model and 10 independent runs.  To obtain bootstrap values, 1000 bootstrap replicates were performed.

*Conflict visualization and identification of rogue taxa*

In order to identify unstable or rogue taxa, we utilized consensus networks in Splitstree [35] to visualize taxa that have conflicting placements in our Bayesian posterior tree distribution. 30,000 trees (15,000 post-stationarity for each of 2 runs) were examined with each consensus network including 1000 to 2000 trees. Threshold was set to zero to include all conflicting splits.  Additionally, in order to create a consensus network representing the entire distribution of each Bayesian run, every 15^th^ tree of each distribution was sampled and 2 consensus networks were constructed from the resulting 1000 trees. Taxa that were found to have multiple attachment points, as seen by reticulation in the network, were then pruned from the posterior distribution. To find posterior probabilities, a majority-rule consensus tree was recalculated without these rogue taxa.  Additionally, we performed a new Bayesian analysis, with the same parameters as the previous analysis, with rogue taxa removed.  

**Results and Discussion**

****

*Molecular findings including all taxa*

**Figure:**
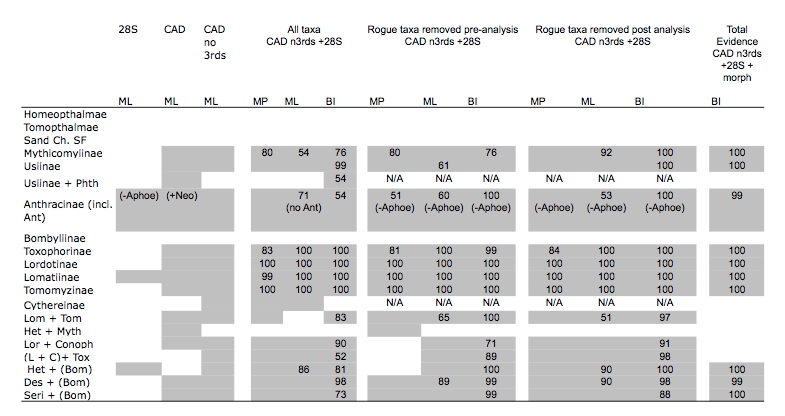
Table 2.  Clade recovery in analyses including all taxa (28S, CAD and combined), with rogue taxa removed pre and post analysis, and total evidence (28S+CAD+morphology).   Single gene analyses do not include support values.  Phth=Phthiria, Ant=Antoniinae, Lom=Lomatiinae, Tom=Tomomyzinae, Het= Heterotropinae, Myth=Mythicomyiinae, Lor= Lordotinae. Conoph= Conophorinae, L+C= Lordotinae + Conophorinae, Tox= Toxophorinae, Des= *Desmatomyia*, Bom= remaining Bombyliidae, Seri= *Sericosoma*, Neo=*Neosardus*

MP, Ml and Bayesian analyses of the combined 28S rDNA (1136 bp) + CAD dataset (2536 bp excluding 3rd positions) yield similar topologies and show high support for the placement of early branching lineages and the monophyly of various tribes and subfamilies (Fig. 3; Table 2). Our trees generally lack strong support for many higher-level groupings and exhibit short branches depicting inter-relationships of bee fly subfamilies.   Out of the 14 subfamilies included in our data set, 9 are represented by more than one taxon and thus were sampled to test for monophyly.  Six of these nine subfamilies were found to be monophyletic in all methods of analysis, including Mythicomyiinae, Toxophorinae, Lomatiinae, Tomomyzinae, Usiinae (sensu Evenhuis 1989 [26]; excluding the Phthiriinae) and Lordotinae.  Under various analysis methodologies, Usiinae (sensu Yeates, 1994 [3]; including Phthiriinae) (BI), Cythereinae (MP and ML) and Anthracinae, at the inclusion of the subfamily Antoniinae (ML and BI), were also found to be monophyletic.  Bombyliinae exhibited far-ranging polyphyly in all methods of analysis.  Tomophthalmae, bee flies with two occipital foramina corresponding to a concave postcranium, Homeophthalmae, and the 'sand chamber subfamilies' were not found to be monophyletic under any method of analysis.

**Figure:**
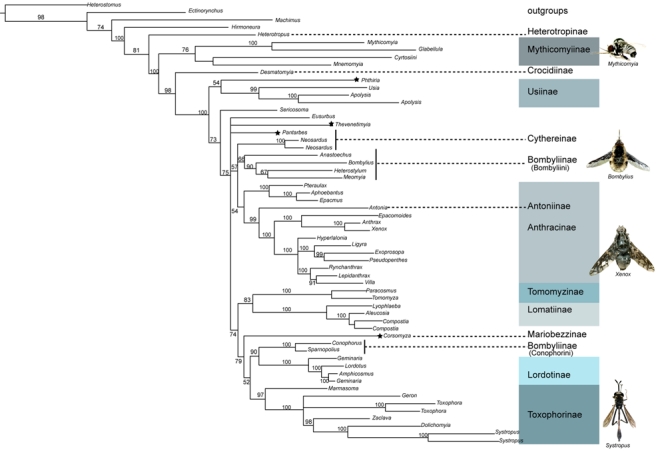
Figure 3. BI analysis of 28S+CAD (3672bp) with 3^rd^ positions removed. GTR+I+G model implemented with genes in independent partitions. 2 runs, 20,000,000 generations with the initial 25% of trees deleted as burn-in. Monophyletic subfamilies are highlighted.  Starred taxa show instability in the posterior distribution and are removed in subsequent analyses to improve support values.

*Molecular analyses with rogue taxa removed*

Though all three methods of analysis primarily agree on the monophyly of the subfamilies, their placement within the family and their relationships to each other varies, often accompanied by low support values. The majority of the incongruence exists between the MP tree and topologies from model-based methods of analysis (ML and BI). When evaluating the phylogeny of ancient radiations such as this one, model-based methods can outperform MP due to their ability to compensate for multiple changes per nucleotide site and rate heterogeneity between sites [36].  Parsimony is more likely to succumb to systematic biases such as long-branch attraction; therefore, conflicting results from MP and model-based methods can indicate such potential systematic biases at work [37] [38] [22] [39].  In further exploratory analyses we relied on Bayesian inference to improve support and resolution for inter-subfamilial relationships, while rectifying artifactual placements.

Unstable or rogue taxa have been shown to have a detrimental effect on phylogenetic recovery and their removal has been shown to increase support values [11] and often correct artifactual misplacements in topologies [39]. Because the effect can be particularly confounding in majority-rule consensus trees [13](Fig 2), the issue of rogue taxa is of increased relevance in reporting results from Bayesian inference (as well as bootstrap values from MP and ML), as this is primarily done using a majority rule consensus tree. A set of relationships prevelant in a posterior distribution of trees, yet disrupted by unstable taxa will remain unresolved or exhibit decreased support in a majority rule consensus tree. In this study, the use of consensus networks allowed us to identify and prune rogue taxa from our final tree distribution, restoring disguised resolution and increased support. 

Consensus networks simultaneously display multiple topologies, revealing conflicting and non-conflicting splits [23].  Examination of consensus networks allowed the visualization of conflicting nodes within the 30,000 trees in our Bayesian posterior distribution and revealed only 4 individual taxa with multiple, differing toplogical placements: *Thevenetimyia*, *Pantarbes*,* Corsomyza*, and *Phthiria* (Fig. 4).  Interestingly, these same taxa have phylogenetic ambiguity in their back history and are members of anomolous subfamilies or tribes.  In our current analyses, *Thevenetimyia*, *Corsomyza*, and *Phthiria* are long-branched. *Corsomyza* and *Phthiria* are the single included representatives of the subfamilies Mariobezzinae and Phthiriinae, respectively.  *Thevenetimyia* is a member of the large, diverse, and, here, polyphyletic, subfamily Bombyliinae, in the morphologically enigmatic tribe Eclimini .  

**Figure:**
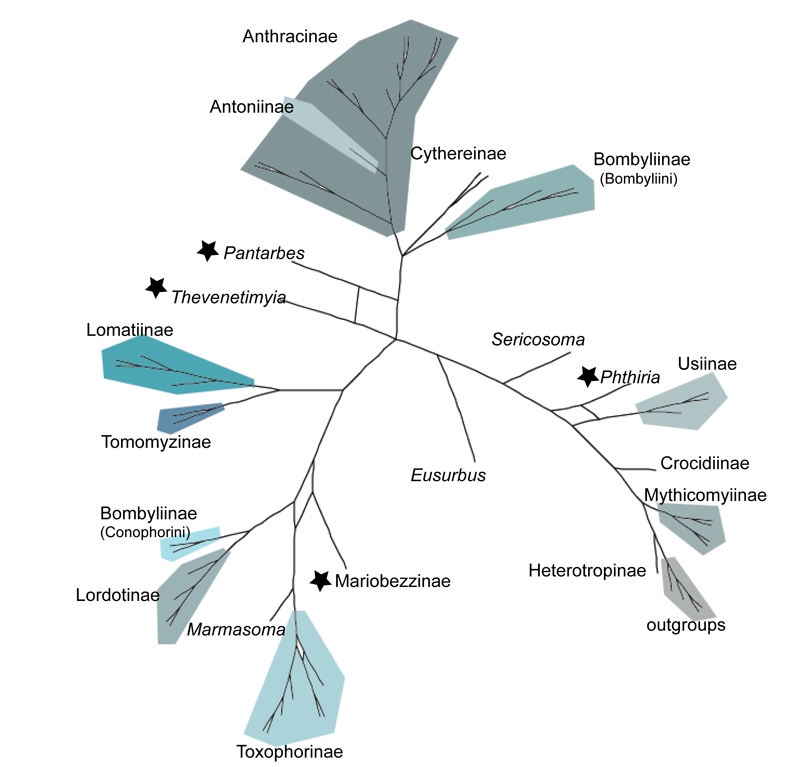
Figure 4.  Consensus network showing the conflicting splits in MP, ML and BI topologies based on 28S+CAD (3672bp) with 3^rd^ positions removed.  Four rogue taxa, noted by stars, have multiple placements shown by node.  In this network, an additional taxon within the Toxophorinae also shows a conflicting split, but because this did not affect the monophyly of the subfamily, it is not considered a rogue taxon. 

Though typical practice is to remove rogue taxa prior to re-analysis, we pruned our rogue taxa post-analysis to allow them to phylogenetically inform the tree, a method supported by simulation studies [24] and recent developed theoretical methodologies addressing rogue taxon removal [13] [15] [12].  The pruning of these 4 taxa from each of the trees within the posterior distribution prior to the construction of a majority-rule consensus tree results in a marked increase in support values for the relationships between bee fly subfamilies (Fig. 5).  Considering the superior performance of model-based methods for old lineages and our ability to identify and remove unstable taxa within the posterior tree distribution, our BI tree with 'rogue' taxa removed is our best current DNA-based estimate of the evolutionary relationships of bee flies (Fig. 6).

**Figure:**
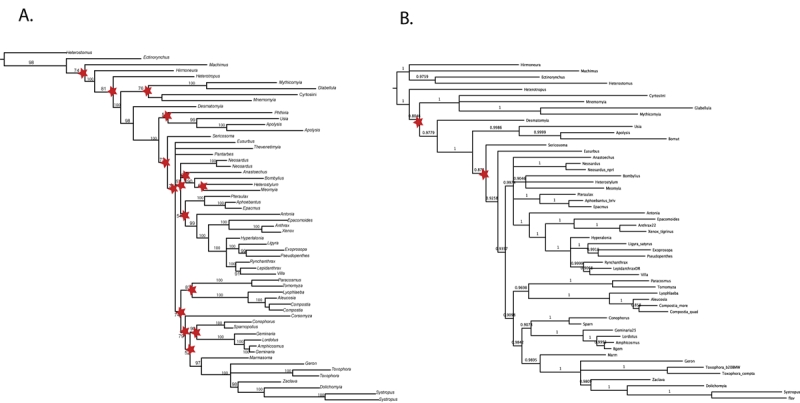
Figure 5.  Pruning 4 rogue taxa from the 30,000 trees in the Bayesian posterior distribution greatly increases posterior probabilities of recovered clades.  Stars indicate nodes with posterior probabilities of less than 90%.  Tree A includes all taxa and tree B has had 4 rogue taxa pruned. After the removal of rogue taxa, 96% of nodes are well-supported (B) , in comparison to 71% prior to removal (A).  This increase in mean branch support is statistically significant ( p= .002).

**Figure:**
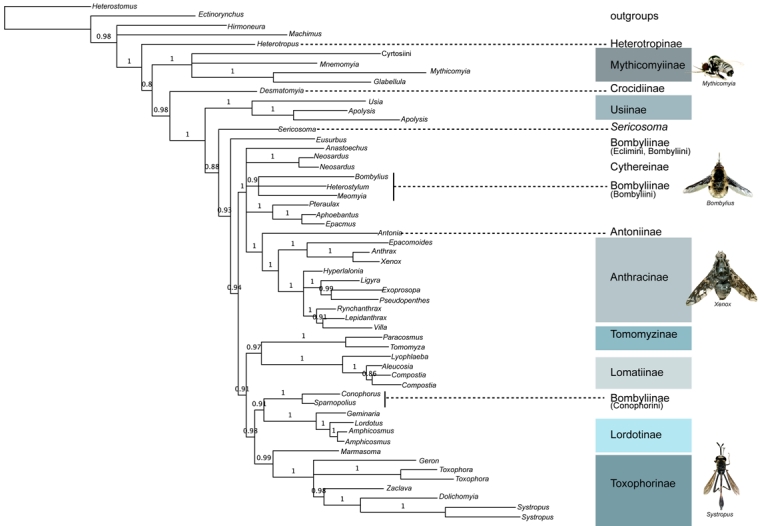
Figure 6. BI analysis of 28S+CAD (3672bp) with 3^rd^ positions removed and GTR+I+G model implemented with both genes in independent partitions. 20,000,000 generations with the initial 25% of trees deleted as burn-in.  Four rogue taxa have been pruned from the posterior distribution prior to construction of the majority-rule consensus.  Monophyletic subfamilies are highlighted. 

Overall, our molecular phylogeny conflicts with the previous morphological estimate, yet both morphology and molecules agree that the microbombyliids, Mythicomyiinae, are an early branching lineage.  Fossil evidence also supports this finding, with microbombyliid fossils appearing millions of years before subsequent bee fly radiations [40] [41] [42].   Additional evidence coroborating the early divergence of micro-bee flies is the fact that they are predators as larvae, unlike most other bee fly subfamilies, and they share this pleisiomorphic life history with other lower brachyceran and asiloid flies.  There has been general consensus that the microbombyliids are the sister group to the remaining bee flies, yet there is disagreement over whether their differences are sufficient for them to be raised to family status as Mythicomyiidae [43] [44] [3] [45]. 

In our molecular phylogeny the earliest diverging lineage is not the Mythicomyiinae, as expected, but instead the monogeneric subfamily Heterotropinae.  Heterotropinae was previously a catch-all subfamily that included misplaced scenopinds and other hard-to-place asiloid-like flies.  It has since been revised and only the namesake genus *Heterotropus* remains [46] [9].  Afrotropical larval *Heterotropus*, identified by Yeates and Irwin (1992) [47], were found to lack the pentultimate larval abdominal spiracle, a synapomorphy unifying all Asiloidea, and thus it was hypothesized that the genus may belong outside of Bombyliidae at the base of the asiloid radiation.  Yeates' 1994 analysis, however, maintained *Heterotropus* within the bee flies in a more derived placement than our data suggest.  An examination of the male genitalia of lower Brachycera by Sinclair and Cumming (1994) [48], found support for *Heterotropus* at the base of the bee fly radiation. *Heterotropus*, like mythicomyiids and in contrast to the rest of Bombyliidae, are minute, enigmatic flies with a predatory larval stage.  These similarities lend additional credence to our well-supported molecular results indicating the ealy divergence of Heterotropinae.

Other well-supported placements at the base of the bee fly tree are that of the small genus *Desmatomyia*, one of two Nearctic genera in the subfamily Crocidinae, and the subfamily Usiinae.  *Desmatomyia *are described by Hull as bare, shining black flies ‘oddly resembling a blackfly gnat, or simuliid’ [1]. Once included in the Bombyliinae, tribe Crocidiini, Yeates placed *Desmatomyia *and the subfamily Crocidinae as the sister group to the division Tomophthalmae.  Our molecular data, however, consistently place the Crocidiinae at the base of the tree in all methods of analysis (MP, ML, BI) with high support.   A point of agreement between our molecular phylogeny and Yeates morphological study is the placement of Usiinae as an early-branching bee fly subfamily.  Yeates’ united the subfamilies Usiinae and Pthiriniinae (a grouping suggested by Hull, 1973 [1], and opposed by Evenhuis and Greathead 1999 [49]), on the basis of (amongst other characters), the similarity of their antennae with subapical and apical styli, respectively.  In our analysis, *Phthria *acted as a rogue taxon and was thus removed from our final tree, although alternate analyses place it both with Usiinae and elsewhere.

In comparison to the ladder of relationships recovered by morphological data, our molecular phylogeny recovers more complex inter-subfamilial relationships.  Our phylogeny reveals two large clades that include the majority of bee fly diversity, with most of Bombyliinae and all of Anthracinae in a single clade (clade 1: Bombyliinae in part, Antoniinae, Cythereinae, and Anthracinae; clade 2: Tomomyzinae, Lomatiinae, Bombyliinae in part, Lordotinae, and Toxophorinae). Some of these higher-level relationships recall previous classifications.  For example, Clade 1 is similar to the polytomy recovered in Yeates’ (1994) [3] reanalysis of the initial cladistic data gathered by Muhlenberg (1971) [7].  Additionally, Anthracinae, Antoniinae and Cythereinae were previously united as part of a monophyletic Tomophthalmae [3].  Clade two introduces some novel relationships between subfamilies, such as the sister-group relationship between Tomomyzinae and Lomatiinae.  This unexpected placement conflicts with the morphology-based hypothesis that Tomomyzinae are the most derived of bee fly subfamilies; however, some morphological features are shared between specific members of Tomomyzinae and Lomatiinae, such as the lack of prealar bristles [3], reinvestigation of these and possibly additional characters may yield new evidence in support of the close relationship reported here.

The large subfamily Bombyliinae is not recovered as a monophyletic assemblage in our analyses.  Yeates states that 'the Bombyliinae itself is a weak clade supported by two apomorphies replete with homoplasy...' [3].   In the World Catalog of Bee Flies, Evenhuis and Greathead express that despite the high degree of homoplasy, 'it is a readily recognizable subfamily- usually robust flies with dense hair on the body and a well developed sand chamber guarded by dense hair.' [49].  Three tribes of Bombyliinae were sampled: Bombyliini, Conophorini, and Eclimini.  None of these tribes were recovered in close vicinity of the other, and only one, Conophorini was found to be monophyletic (though 3 out of 5 members of the tribe Bombyliini also generally formed a clade).  It is notable that the tribe Conophorini was found to be the sister-group to Lordotinae, a subfamily whose included genera were previously members of the tribe Conophorini before being raised to subfamily status by Yeates' morphological revision [3].  Lordotinae and Conophorini share some morphological features, including similarities in wing venation as well as the possesion of one midtibial spur [3], and our molecular data indicate that there is evidence for considering their reunification.  The tribe Eclimini was represented by *Thevenetimyia, *a rogue taxon removed from our final phylogeny, and *Marmasoma*, an Australian genus interestingly placed as the sister-group to Toxophorinae, the subfamily in which it was previously included [1].  

Anthracinae, the most species-rich subfamily of bee flies, are well supported as a monophyletic group in our final phylogeny (including the tribes Anthracini, Exoprosopini, and Villini) at both the tribe and subfamily level, with the single taxon representative of the subfamily Antoninae as their sister group (Fig 6).  The tribe Aphoebantini, separates itself from the remainder of the subfamily and is part of the polytomy of clade 1.  Yeates’ morphological data also supported the Anthracini, Exoprosopini and Villini as a monophyletic group and he points out that the placement of the remaining anthracine tribes, including Aphoebantini, is less clear [3].  The Aphoebantini have a history of phylogenetic ambiguity, having been previously placed in the Lomatiinae [1].

Tomophthalmae and the 'sand chamber subfamilies', both previously considered monophyletic groups, are not recovered in our analyses. Our phylogeny indicates that the morphological characters that defined these two groups, the possesion of two occipital foramina and the sand chamber, exhibit a great deal of plasticity in gain and loss across subfamily-level divergences.  The 'sand chamber subfamilies' as defined by Yeates (1994) [3], already included apomorphic instances of secondary loss of this female genetalic character, for example, in members of Antoniinae. Evenhuis and Greathead's 'phylogenetic considerations' in the World Catalog of Bee Flies (1999) [49] also addresses the variations and modifications of the sand chamber amongst the members of the 'sand chamber subfamilies'.  Homplasy was already evident within this group, yet the scale of the homoplasy, now seen across virtually the entire family, will have to be revisited.  

*Congruence, branch lengths, and future work*

Though there is little topological congruence between our molecular results and the morphological results from the only other quantitative phylogeny for bee flies, there is a common pattern that both molecular and morphological data yield short branches in the backbone (internal divergences) in all tree estimates.  28S alone and in combination with CAD has been shown to provide strong resolution to many areas of the fly tree of life [50] [51] [52] [53] [54] [55], however these genes have not been as effective for the bee flies and their superfamily, Asiloidea [5]. Morphological and molecular analyses have established that the evolutionary relationships in this region of the fly tree are challenging to resolve with robust support[53] [5]] [56] [4]. Ancient radiations, such as the bee flies, can be difficult to recover with limited amounts of data, particularly if taxon diversification took place in rapid succession leaving few polarizing characters to discern the relationships between lineages [57] [58] [36]. Though our short branches may be an accurate reflection of evolutionary history [36], future work will be required to test the resolving power of additional genes to our data set.  Additionally, we will look to increased taxon sampling to break up the long branches of often single representatives of enigmatic lineages in our current study [59] [60].  This may help determine the accurate placement of *Sericosoma*, Mariobezzinae, and the Bombyliinae tribe Eclimini, amongst others.  

****

**Conclusion**

Our molecular study of the evolutionary relationships between the subfamilies of the bee flies has resulted in the confirmation of the monophyly of eight of fifteen subfamilies with varying levels of support, in addition to demonstrating wide-ranging polyphyly of the large subfamily Bombyliinae.  Our findings indicate a need to reevaluate inferences concerning the evolution and plasticity of key morphological characters previously used to define the now polyphyletic divisions Tomophthalmae and the sand chamber subfamilies.  We confirm, in consensus with morphology and fossil data, that Mythicomiinae is an early branching lineage of bee flies, while Heterotropinae is found to be the earliest lineage, sister to the remaining bee flies. We find that the utilization of consensus networks to identify rogue taxa and their removal post-analysis greatly increases support values for recovered clades.   In conclusion, we provide a molecular foundation for future work on Bombyliidae, along with a new phylogenetic hypothesis to guide and direct alternative lines of inquiry for this diverse family of flies.

**Acknowledgements**

We are especially grateful to our colleagues M.E. Irwin, F. Lamas, S. L. Winterton, A. Friedberg, T. Dikow and N.I. Evenhuis for the provision and identification of specimens.  Thanks to B. Redelings for discussions of methods for dealing with rogue taxa.  Additionallly, grateful thanks to Brian K. Cassel  (NCSU) for assistance in the collection of molecular data, the NCSU Genomic Sciences Laboratory (GSL) for DNA sequencing. This project was supported by US National Science Foundation (NSF) Assembling the Tree of Life (ATOL) grant EF-03394 to BMW and DKY.

**Competing Interests**

The authors have declared that no competing interests exist.
